# Molecular characteristics of oxazolidinone resistance in enterococci from a multicenter study in China

**DOI:** 10.1186/s12866-019-1537-0

**Published:** 2019-07-12

**Authors:** Hongbin Chen, Xiaojuan Wang, Yuyao Yin, Shuguang Li, Yawei Zhang, Qi Wang, Hui Wang

**Affiliations:** Department of Clinical Laboratory, Peking University People’s Hospital, Beijing, 100044 People’s Republic of China

**Keywords:** *optrA*, Linezolid resistance, Oxazolidinone, Enterococci, Genetic environment

## Abstract

**Background:**

Linezolid-resistant enterococci pose great challenges in clinical practice. The aim of this study is to study the mechanisms underlying the resistance and genetic environment of antimicrobial resistance gene of linezolid-resistant enterococci.

**Results:**

The linezolid MICs of 16 enterococci were 4 mg/L to 16 mg/L. Four strains belonged to multi-drug resistant (MDR) bacteria. The sequence types (STs) of 13 enterococci strains performed WGS were diverse: 3 ST476, 1 ST86, ST116, ST480, ST59, ST416, ST21, ST67, ST16, ST585 and ST18. None of them carried multi-drug resistance gene *cfr*. Only one strain had the G2658 T mutation of target 23S rRNA gene. Thirteen (13/16, 81.3%) strains harbored the novel oxazolidinone resistance gene *optrA*. WGS analysis showed that the *optrA* gene was flanked by sequence IS*1216E* insertion in 13 strains, and *optrA* was adjacent to transposons Tn*558* in two strains and Tn*554* in one strain. The *optrA* gene was identified to be co-localized with *fexA,* the resistance genes mediated florfenicol resistance in 13 strains, and *ermA1,* the resistance genes mediated erythromycin resistance in 9 strains, indicating that linezolid-resistant strains may be selected due to non-oxazolidinone antibiotics (i.e. macrolides and florfenicol) usage.

**Conclusion:**

Our findings demonstrate the high diversity of *optrA*-carrying genetic platforms. The mobile genetic elements (MGEs) may play an important role in the dissemination of *optrA* into the enterococci isolates of human origin. The genetic evidence of transferable feature and co-selection of *optrA* should be gave more attention in clinical practice.

## Background

Linezolid, which belongs to oxazolidinone, is the clinically last resort to treat vancomycin-resistant enterococci (VRE), methicillin-resistant *Staphylococcus aureus* (MRSA), and other multi-drug Gram-positive bacteria [1]. Linezolid exerts antibacterial effects by inhibiting the binding of mRNA to the ribosome, thereby affecting the synthesis of the protein [1]. It is generally considered that linezolid is a completely synthetic antibiotic, and theoretically, there should be no natural resistance phenomenon. Unfortunately, clinically resistant strains have emerged shortly after use of linezolid in clinical practice [2, 3]. The occurrence of linezolid-resistant strains show an increasing trend, especially in animal husbandry [4], which should attract sufficient attention.

The resistance to linezolid by gram-positive bacteria can be achieved by target-modified 23S rRNA mutations [5], acquiring exogenous chloramphenicol-florfenicol resistance (*cfr*) [6], *optrA* [7] or *poxtA* [8]. Targets 23S rRNA, L3, L4 and L22 mutations usually affect ribosome function and easily reverse in the absence of selective pressure. Therefore, chemical modifications (such as methylation) of rRNA are the more common resistance mechanisms of linezolid. The *cfr* gene encodes a methyltransferase that modifies the 23S rRNA at position A2503, which confers resistance to phenicols, lincosamide, oxazolidinones, pleuromutilin, and streptogramin A (PhLOPS_A_ phenotype) [9]. The *cfr* gene has been identified in a variety of genera, including *Staphylococcus* [10], *Bacillus* [11], *Enterococcus* [12], *Macrococcus* [13], *Jeotgalicoccus* [13], *Streptococcus* [14], *Proteus* [15] and *Escherichia* [16]. The *cfr* gene widely disseminates among oxazolidinone-resistant isolates from human [17] and animal [18] origin, which represents a serious threat to public health. Recently, two *cfr* variants, *cfr*(B) and *cfr*(C), have been found in *Enterococcus faecium* [19], *Clostridium difficile* [20] and *Campylobacter* [21]. The *cfr* gene was often found on a number of different plasmids [7, 15, 22], and integrated into transposons, leading to dissemination of this gene among the same or between different species of bacteria.

The transferable gene, *optrA*, has been identified, which confers cross-resistance to phenicols and oxazolidinones, including tedizolid [23]. This gene was identified in enterococci and staphylococci from clinical [24], healthy human and animal isolates [25, 26]. The resistance gene *optrA* can be located either on plasmid or chromosome [26]. Recently, one florfenicol-resistant *Staphylococcus sciuri* isolate, which carried both *optrA* and *cfr*, was identified in pig [27]. In this study, we investigated the oxazolidinones resistance genes among linezolid-resistant isolates in Chinese hospitals and utilized whole-genome sequencing (WGS), and further analyzed the genetic environment surrounding the resistance genes.

## Materials and methods

### Bacterial strains

A total of 15 non-duplicable linezolid-resistant enterococci strains and one linezolid intermediate-resistant enterococci strain (13 *E. faecalis* and 3 *E. faecium*) (1.5%, 16/1067) were collected from specimens of 16 patients from 9 hospitals between 2009 and 2013 in 6 provinces of China, including 5 samples from Beijing, 4 samples from Guangdong, 3 samples from Zhejiang, 2 samples from Fujian, 1 sample from Jiangsu and 1 sample from Hubei (Table 1.). Among the 16 strains, 6 were recovered from patients with urinary tract infection, 5 from patients with bacteremia, 4 from patients with wound infection and 1 from patients with biliary tract infection. Among the 16 strains, 7 strains (1203_10W003, 1202_13E004, 1202_21W014, 19113, 19677, 19506 and SZ21494) were isolated in our previous study [28], and the 9 remaining strains were isolated in this study. Bacteria were first identified at the species level using the VITEK system (bioMerieux, Crapome, France), followed by a molecular method based on the 16S rRNA gene, and then by sequencing analysis.

**Table 1:** Clinical, phenotypic and genotypic data for the linezolid-resistant Enterococci isolates investigated

Isolate no.	Organism	Isolation year	Hospital^b^	Isolation site	ST^c^	MICs (mg/L)^a^											Linezolid resistance genes	23S rRNA gene mutations	Other resistance genes
						LNZ	P	AMP	VAN	TEC	DAP	TGC	LVX	ERY	HLG	Antibiotic resistance profiles			
29462	*E. faecalis*	2009	ZRYH	urine	86	8	4	<=2	2	<=0.125	0.5	0.06	8	> 4	R	LVX, ERY	*optrA*	–	*emeA, ANT(6)-Ia, AAC(6′)-Ie-APH(2″)-Ia, dfrG, dfrE, lsaA, fexA, cat, efrB, efrA, ermB, tetM, tet(L)*
ZJ11066	*E. faecalis*	2011	ZJFY	blood	116	8	2	<=2	1	0.125	0.5	0.12	8	> 4	R	LVX, ERY	*optrA*	–	*emeA, APH(3′)-IIIa, AAC(6′)-Ie-APH(2″)-Ia, dfrF, dfrG, dfrE, lnuG, lsaA, fexA, efrA, efrB, ermB, ermA1, tet(L), tetM*
JS11041	*E. faecium*	2011	JSRM	urine	ND	8	> = 64	> = 32	0.5	0.25	2	0.06	8	> 4	R	P, AMP, LVX, ERY	*–*	–	ND
19113	*E. faecalis*	2011	SZRM	bile	ND	8	> = 64	> = 32	2	<=0.125	0.5	0.06	1	> 4	R	P, AMP, LVX, ERY	*–*	–	ND
ZJLRE1	*E. faecium*	2011	ZJFE	blood	ND	16	> = 64	> = 32	1	0.5	1	0.06	8	> 4	R	P, AMP, LVX, ERY	*–*	G2658 T	ND
1207_26W003	*E. faecalis*	2012	BJRM	urine	476	4	2	<=2	1	0.12	0.5	0.06	8	> 4	R	LVX, ERY	*optrA*	–	*emeA, APH(3′)-IIIa, AAC(6′)-Ie-APH(2″)-Ia, aad(6), ANT(9)-Ia, dfrG, dfrE, lnuB, lsaE, lsaA, mdtF, SAT-4, cat, fexA, efrB, efrA, ermA1, ermB, tet(L), tetM*
1203_10W003	*E. faecalis*	2012	BJRM	urine	480	8	2	<=2	1	0.12	0.5	0.06	8	> 4	R	LVX, ERY	*optrA*	–	*emeA, AAC(6′)-Ie-APH(2″)-Ia, APH(3′)-IIIa, aad(6), ANT(6)-Ia, dfrG, dfrE, lnuB, lsaE, lsaA, SAT-4, cat, fexA, efrA, efrB, ermB, ermA1, tetM, tet(L)*
19677	*E. faecalis*	2012	SZRM	blood	59	8	2	<=2	0.5	0.12	0.5	0.12	0.03	> 4	R	ERY	*optrA*	–	*emeA, dfrE, lsaA, fexA, efrA, efrB, ermA1, tetM, tet(L)*
19506	*E. faecium*	2012	SZRM	wound	18	16	> = 64	> = 32	0.5	0.25	2	0.06	8	> 4	S	P, AMP, LVX, ERY	*optrA*	–	*AAC(6′)-Ii, dfrG, efmA, msrC, fexA, ermA1*
1202_13E004	*E. faecalis*	2012	BJRM	wound	416	16	8	<=2	2	<=0.125	0.5	0.12	8	> 4	R	LVX, ERY	*optrA*	–	*emeA, ANT(6)-Ia, AAC(6′)-Ie-APH(2″)-Ia, dfrG, dfrE, lsaA, fexA, efrB, efrA, ermB, ermA1, tet(L), tetM*
1202_21W014	*E. faecalis*	2012	BJRM	urine	21	8	4	<=2	2	<=0.125	0.5	0.12	8	> 4	R	LVX, ERY	*optrA*	–	*emeA, AAC(6′)-Ie-APH(2″)-Ia, aad(6), ANT(6)-Ia, dfrG, dfrE, lnuG, lsaA, SAT-4, fexA, cat, efrA, efrB, ermB, tet(L)*
SZ21494	*E. faecalis*	2012	SZRM	wound	67	8	4	<=2	1	<=0.125	1	0.06	1	> 4	S	ERY	*optrA*	–	*emeA, dfrE, dfrG, lnuG, lsaA, fexA, cat, efrA, efrB, ermB, ermA1, tetM, tet(L)*
XM2013_71028	*E. faecalis*	2013	XMDY	wound	16	8	2	<=2	1	<=0.125	1	0.06	0.5	> 4	R	ERY	*optrA*	–	*emeA, APH(3′)-IIIa, AAC(6′)-Ie-APH(2″)-Ia, ANT(9)-Ia, aad(6), dfrG, dfrE, lnuB, lsaE, lsaA, SAT-4, fexA, cat, efrB, efrA, ermB, ermA1, tetM*
XM2013_42321	*E. faecalis*	2013	XMDY	urine	585	16	4	<=2	1	<=0.125	0.5	0.06	8	> 4	R	LVX, ERY	*optrA*	–	*emeA, APH(3′)-IIIa, AAC(6′)-Ie-APH(2″)-Ia, aad(6), ANT(9)-Ia, dfrE, dfrG, lmrD, lnuB, lsaE, lsaA, SAT-4, cat, fexA, efrB, efrA, ermB, tetM, tet(L)*
TZ2	*E. faecalis*	2013	TZSY	blood	476	8	2	<=2	1	<=0.125	0.5	0.12	8	> 4	R	LVX, ERY	*optrA*	–	*emeA, AAC(6′)-Ie-APH(2″)-Ia, APH(3′)-IIIa, aad(6), ANT(6)-Ia, dfrG, dfrE, lsaA, SAT-4, fexA, cat, efrB, efrA, ermA1, ermB, tet(L), tetM*
WHXH	*E. faecalis*	2013	WHDS	blood	476	8	4	<=2	2	<=0.125	0.5	0.12	8	> 4	S	LVX, ERY	*optrA*	–	*emeA, AAC(6′)-Ie-APH(2″)-Ia, APH(3′)-IIIa, aad(6), ANT(9)-Ia, dfrE, dfrG, lnuB, lsaE, lsaA, SAT-4, cat, fexA, efrB, efrA, ermB, tetM, tet(C), tet(L)*

^a^MICs, the minimal inhibitory concentrations; LNZ, linezolid, susceptible (S): ≤ 2 mg/L, intermediate (I): 4 mg/L, resistant (R): ≥ 8 mg/L; P, penicillin, S: ≤ 2 mg/L, R: ≥ 8 mg/L; AMP, ampicillin, S: ≤ 2 mg/L, R: ≥ 8 mg/L; VAN, vancomycin, S: ≤ 4 mg/L, I: 8–16 mg/L, R: ≥ 32 mg/L; TEC, teicoplanin, S: ≤ 8 mg/L, I: 16 mg/L, R: ≥ 32 mg/L; DAP, S: ≤ 1 mg/L, susceptible-dose dependent (SDD): 2–4 mg/L, R: ≥ 8 mg/L; TGC, tigecycline, no breakpoint in CLSI M100; LVX, levofloxacin, S: ≤ 2 mg/L, I: 4 mg/L, R: ≥ 8 mg/L; ERY, erythromycin, S: ≤ 0.5 mg/L, I: 1–4 mg/L, R: ≥ 8 mg/L; HLG, high-level gentamycin (500 mg/L); −, negative; ND, not determined

^b^ZRYH, China-Japan Friendship Hospital; ZJFY, 1st Affiliated Hospital of Zhejiang University; JSRM, Jiangsu Province Hospital; SZRM, Shenzhen People’s Hospital; ZJFE, 2nd Affiliated Hospital of Zhejiang University; BJRM, Peking University People’s Hospital; XMDY, 1st Affiliated Hospital of Xiamen University; TZSY, Taizhou Hospital of Zhejiang Province; WHDS, Wuhan Fourth Hospital

^c^*ST* sequence type, *ND* not determined

### Antimicrobial susceptibility testing

The minimal inhibitory concentrations (MICs) of 8 antimicrobial agents were determined by the agar dilution method, and tigecycline and daptomycin by broth microdilution. The antimicrobial agents tested included linezolid (Sigma Chemical Co., St. Louis, MO, USA), vancomycin (Sigma), teicoplanin (Sigma), levofloxacin (Sigma), erythromycin (Sigma), tigecycline (Pfizer, NY, USA), daptomycin (Cubist Pharmaceuticals, MA, USA), penicillin (Sigma), ampicillin (Sigma) and gentamycin (Sigma). *E. faecalis* ATCC 29212 was used for quality control in antimicrobial susceptibility testing. The results of susceptibility testing were interpreted according to CLSI guideline M100-S27. Isolates resistant to three or more antibiotics of different families were considered to be multi-drug resistant (MDR).

### Molecular detection of resistance genes and mutations

The resistance genes *cfr* and *optrA* were determined by PCR as described previously. The mutation of domain V of the 23S rRNA gene was determined by PCR combined with sequencing as described previously [29]. Nucleotide sequences were compared with the linezolid-susceptible *E. faecalis* and *E. faecium* from Peking University People’s Hospital during the same period. The mutation was identified by the *E. coli* numbering.

### Whole-genome sequencing (WGS)

Total genomic DNA of 13 enterococci strains carrying *optrA* gene was extracted by the standard phenol/chloroform method. The whole-genome sequencing was performed using Illumina technology. The sequences with read length of 150 bases were assembled into contigs using SPAdes (v.3.9.0) [30]. Plasmid content associated with *optrA* was analyzed using the contigs obtained by plasmidSPAdes. The assembled contigs were annotated by the Prokka v1.12 [31]. Insertion sequences (IS) were identified using ISFinder [32]. Multilocus sequence types (MLST) were assigned using the silico tool hosted by Center for Genomic Epidemiology (CGE) (www.genomicepidemiology.org). The resistance genes were identified by ResFinder 3.0 [33]. Maximum likelihood phylogenetic analysis of the core genome was performed using RAxML (Linux version v7.2.8) [34]. The sequences of the *optrA*-containing regions of 13 enterococci strains have been deposited at GenBank under the following accession numbers MH225413 (1202_13E004), MH225414 (1202_21W014), MH225415 (1203_10W003), MH225416 (1207_26W003), MH225417 (19506), MH225418 (19677), MH225419 (29462), MH225420 (SZ21494), MH225421 (TZ2), MH225422 (WHXH), MH225423 (XM2013_42321), MH225424 (XM2013_71028) and MH225425 (ZJ11066).

## Results

### Susceptibility profiles of linezolid-resistant enterococci isolates

The susceptible breakpoint of enterococci to linezolid is defined as less than or equal to 2 mg/L, and the resistant breakpoint is defined as greater than or equal to 8 mg/L. The linezolid MICs of 16 enterococci were 4 mg/L to 16 mg/L, respectively. There were no significant differences in the linezolid MICs between *optrA*-positive strains (4–16 mg/L) and *optrA*-negative strains (8–16 mg/L). Most of the *optrA*-positive strains also exhibited resistance to erythromycin (16/16, 100%), levofloxacin (12/16, 75%) and high-level gentamycin (500 mg/L) (13/16, 81.3%). All strains were susceptible to vancomycin, teicoplanin, daptomycin and tigecycline. Three *E.faecium* and one *E. faecalis* strains (4/16, 25%) were resistant to penicillin and ampicillin, and all of 16 enterococci strains didn’t possess beta-lactamase. Four strains (4/16, 25%) belonged to MDR organism (Table 1).

### Distribution of antimicrobial resistance genes

None of 16 linezolid-resistant enterococci strains contained *cfr* gene. Only one strain had the G2658 T mutation in 23S rRNA gene with linezolid MIC of 16 mg/L. Most of the linezolid-resistant enterococci strains (*n* = 13) carried *optrA* gene (Table 1).

In addition to *optrA* genes, all *optrA*-positive strains harbored phenicols resistance gene *fexA* (13/13, 100%), erythromycin resistance genes of different *erm* gene classes (*ermA1, ermB*) (13/13, 100%), trimethoprim resistant dihydrofolate reductase different *dfr* gene classes (*dfrE, dfrG*) (13/13, 100%), ATP-binding cassette (ABC) antibiotic efflux pump different gene classes (*lsaA*, *lsaE*, *efrA*, *efrB*) (13/13, 100%). Further, majority *optrA*-positive strains carried tetracycline resistance genes of different *tet* gene classes (*tet[C]*, *tet[L]*, *tetM*) (12/13, 92.3%), multidrug and toxic compound extrusion (MATE) transporter *emeA* gene (12/13, 92.3%) and aminoglycosides inactivating enzyme different gene classes (*AAC(6′)-Ii*, *AAC[6′]-Ie-APH[2″]-Ia*, *APH[3′]-IIIa*, *aad* [6], *ANT[6]-Ia*, *ANT[9]-Ia*) (10/13, 76.9%). Various additional resistance genes were identified including *cat*, *lnuB*, *lnuG*, *mdtF*, *SAT-4* and *efmA*.

### Core-genome phylogenetic analysis

The 12 *E. faecalis* isolates performed WGS were classified into 10 sequence types (STs): 3 ST476, 1 ST86, ST116, ST480, ST59, ST416, ST21, ST67, ST16 and ST585, respectively. One *E. faecium* isolate belonged to ST18.

**Fig. 1:**
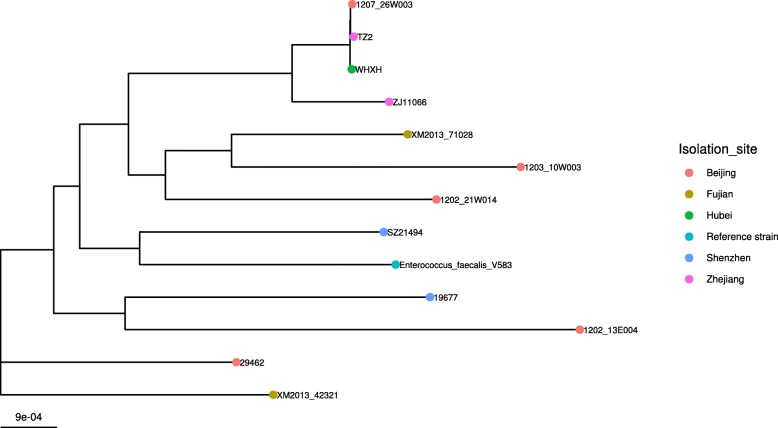
Maximum-likelihood phylogenetic tree of *E. faecalis* (*n* = 12)

The phylogenetic tree of 12 *E. faecalis* isolates harboring *optrA* gene showed that two of these isolates (29462 and XM2013_42321) were genetically unrelated with the rest isolates. Importantly, 1207_26W003 (Beijing), TZ2 (Zhejiang) and WHXH (Hubei) were recovered from different cities, were found very closely related (99.9%), and all of 3 strains belonged to ST476. In addition, strain 19677 recovered from Guangdong was closely related (99.4%) to strain 1202_13E004 recovered from Beijing. Further, strain 1203_10W003 isolated from Beijing and strain XM2013_71028 isolated from Fujian was closely related (99.3%) (Fig. 1).

### Genetic environment of *optrA* on plasmids or chromosome

**Fig. 2:**
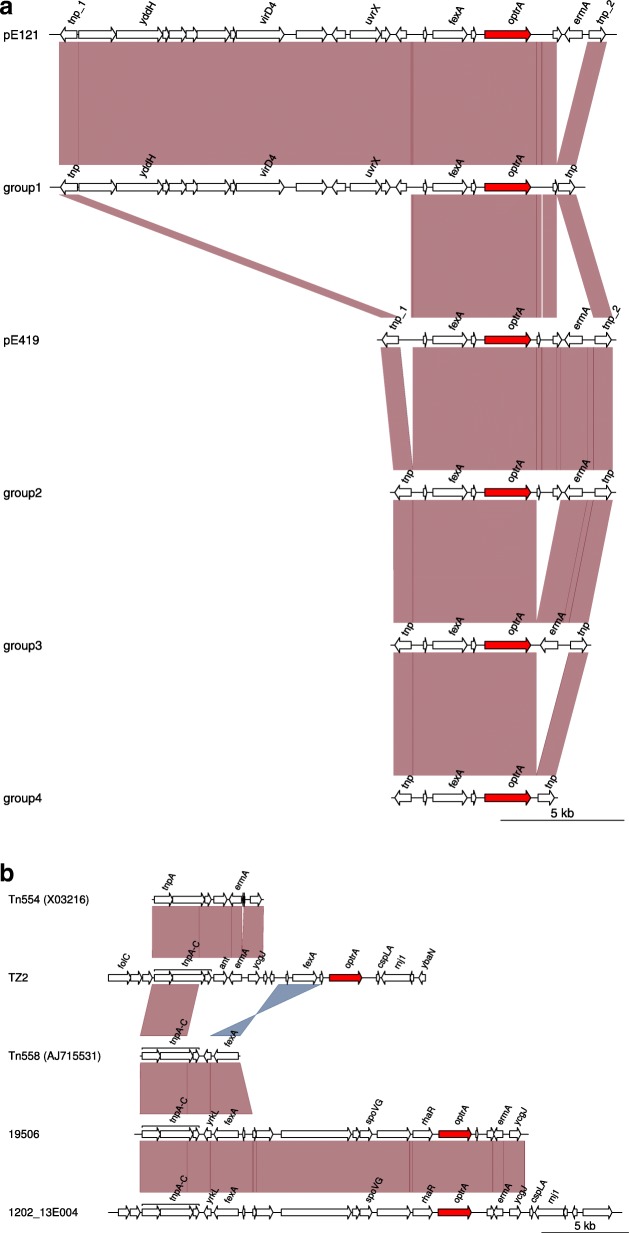
**a** Schematic presentation of the genetic environment of *optrA-*containing contigs mapped on plasmids in 10 enterococci isolates investigated in this study. **b** Schematic presentation of *optrA*-containing contigs mapped on chromosome in three enterococci isolates. Arrows indicate the positions and directions of transcription of the different genes. Genes with unknown functions are not marked. According to the gene arrangement, the 10 contigs mapped on plasmids were divided into 4 groups-group 1 (29462 [MH225419], 1202_21W014 [MH225414]), group 2 (1203_10W003 [MH225415], SZ21494 [MH225420], ZJ11066 [MH225425]), group 3 (1207_26W003 [MH225416], 19677 [MH225418], XM2013_71028 [MH225424]), group 4 (WHXH [MH225422], XM2013_42321 [MH225423])

Thirteen contigs containing the *optrA* gene were blasted in the GenBank database, and 10 contigs were mapped against the plasmids (pE121 [GenBank accession number KT862776] and pE419 [KT862777]). The size of these 10 contigs was between 6372 bp and 21568 bp. According to the gene arrangements, the 10 contigs were divided into 4 groups: group 1 (29462 [MH225419], 1202_21W014 [MH225414]), group 2 (1203_10W003 [MH225415], SZ21494 [MH225420], ZJ11066 [MH225425]), group 3 (1207_26W003 [MH225416], 19677 [MH225418], XM2013_71028 [MH225424]), group 4 (WHXH [MH225422], XM2013_42321 [MH225423]). The genetic environment of *optrA* in Group 1 was similar to that of plasmid pE121 (KT862776). Compared to the plasmid pE121, *ermA1* gene was absent and the rest of the sequences were almost identical. The genetic environment of *optrA* from Group 2 to Group 4 resembled that of plasmid pE419 (KT862777). Compared with pE419, the intergenic region between the left IS*1216E* and the first hypothetical protein was truncated in Group 2, two hypothetical proteins between *optrA* gene and the right IS*1216E* were missing in Group 3, and *ermA1* gene and two hypothetical proteins were missing in Group 4. The common feature of genetic environment of *optrA* from Group 1 to Group 4 was flanked by IS*1216E,* and all of them carried phenicol resistance gene *fexA* and erythromycin resistance gene *ermA1* (Fig. 2a.)*.*

The contigs containing *optrA* gene of 1202_13E004 (MH225413) (29141 bp), 19506 (MH225417) (22720 bp) and TZ2 (MH225421) (75117 bp) were mapped on chromosomal (CP008816). The strains 1202_13E004 and 19506 contained a transposon Tn*558* (AJ715531) with three transposases and the resistance gene *fexA*, and the resistance gene *optrA* was adjacent to resistance gene *ermA1*. The strain TZ2 carried another transposon Tn*554* (X03216) with three transposases and the resistance gene *ermA1*, and *optrA* was adjacent to resistance gene *fexA* (Fig. 2b.).

## Discussion

This study indicates that the transferable resistance gene *optrA* is very prevalent among linezolid-resistant enterococci strains isolated from human. Much more *optrA* gene is located on plasmid than chromosome. The *optrA* gene located on plasmid is flanked by IS*1216E*, while that located on chromosome is mediated by transposons.

In this study, none of linezolid-resistant enterococci strains carried *cfr*, while most of them harbored *optrA*. This suggests that acquiring *optrA* is the main resistant mechanism in linezolid-resistant enterococci from human origin. The presence of *optrA* was limited to a few species of the genus *Enterococcus* [35] and only rare species of *Staphylococcus* [4]. The surveillance studies indicated that only 3.9–6.2% of staphylococci strains were positive for *optrA* [4, 25], which suggests a low prevalence of this oxazolidinone resistance gene in the genus *Staphylococcus*.

In present study, the *optrA* gene was located on plasmids in most of enterococci strains. The *optrA* gene is often surrounded by insertion sequences when located on plasmids from enterococci strains. Our data showed that all of *optrA* found on plasmids were flanked by IS*1216E*, which was similar to a previous study [26]. Other studies also found that co-localization of *optrA* and *cfr* was close to IS*21–558* and IS*257* in *S. sciuri* [4, 27]. IS*1216E* belongs to the IS*6* family which among other mediates transmission of the vancomycin resistance gene *vanA* in *E. faecium*, the oxazolidinone resistance gene *cfr* in *E. faecalis* [36], the macrolide-lincosamide-streptogramin B resistance genes *erm(B)* and *erm(T)* in *E. hirae* [37] and *Streptococcus gallolyticus subsp. pasteurianus* [38], respectively, and the tetracycline resistance gene *tet(S)* in *Streptococcus infantis* [39]. This indicates that *optrA* can be transferred between different genus bacteria by IS-mediated recombination events. Our study found that the *optrA* gene was located on chromosome in a few of enterococci strains. The *optrA* gene was adjacent to transposon Tn*558* in two strains and to Tn*554* in one strain. Tn*558* was also detected upstream of *optrA* gene in *S. sciuri* and *E. faecalis*. The functionally active Tn*558* and Tn*554* could excise from their host DNA and produce circular forms which precede the integration of the transposon into a new target sequence [40]. The similar genetic arrangement of Tn*554* and *optrA* was identified in both of staphylococci and enterococci, which suggest *optrA* can be disseminated mediated by transposon between different genus bacteria. The *optrA* gene was flanked by insertion sequences or transposons, indicating that mobile genetic elements mediate horizontal transfer of *optrA* among different genus bacteria, which should be given more attention to avoid this novel oxazolidinone resistance gene dissemination in hospitals.

Our data showed the co-localization of resistance genes *fexA* (*n* = 13) and *ermA1* (*n* = 9) with *optrA*. The gene *fexA* mediates resistance to fluorinated and non-fluorinated phenicols, which are widely used in livestock, but not in humans. The *fexA* gene was prevalent in florfenicol-resistant staphylcococci [4] and enterococci [23] from animal origin. The evidence of co-localization of *fexA*, *ermA1* and *optrA* indicates that linezolid-resistant strains may be selected due to non-oxazolidinone antibiotics usage, such as macrolides (often used in hospital), florfenicol (often used in livestock) and et al.. The widespread use of florfenicol in livestock has exerted selective pressure on environmental bacteria and poses a significant public health threat to the increased resistance of the novel antibiotic linezolid.

In summary, *optrA* was found in most of linezolid-resistant enterococci. The high diversity of *optrA*-carrying genetic platforms was found even in a limited number of analyzed isolates. The role of *optrA* in enterococci resistance to linezolid requires further investigation. The *optrA* gene was often flanked by insertion sequences or transposons, which might mediate the spread of *optrA* between different species or strains. The co-localization of *fexA*, *ermA1* and *optrA* suggests that linezolid-resistant enterococci can be selected by other antibiotics such as macrolides and so on, which should be given more attention in clinical practice.

## Conclusion

We discovered the high diversity of *optrA*-carrying genetic platforms in our limited number of analyzed isolates. MGE mediated the dissemination of *optrA* between different species or strains. The *optrA* gene was found in most of the linezolid-resistant enterococci. Further studies should be done to clarify the linezolid resistance mechanism of *optrA* gene in Enterococcus species.

## Data Availability

The sequences of the *optrA*-containing regions of 13 enterococci strains have been deposited at GenBank under the following accession numbers MH225413 (1202_13E004), MH225414 (1202_21W014), MH225415 (1203_10W003), MH225416 (1207_26W003), MH225417 (19506), MH225418 (19677), MH225419 (29462), MH225420 (SZ21494), MH225421 (TZ2), MH225422 (WHXH), MH225423 (XM2013_42321), MH225424 (XM2013_71028) and MH225425 (ZJ11066).

## Abbreviations

ABC: ATP-binding cassette
*cfr*: chloramphenicol-florfenicol resistance
IS: Insertion sequences
MATE: Multidrug and toxic compound extrusion
MDR: Multi-drug resistant
MGE: Mobile genetic element
MICs: Minimal inhibitory concentrations
MRSA: Methicillin-resistant *Staphylococcus aureus*
PhLOPS_A_: Phenicols, lincosamide, oxazolidinones, pleuromutilin, and streptogramin A
VRE: Vancomycin-resistant enterococci
VRSA: Vancomycin-resistant *Staphylococcus aureus*
WGS: Whole-genome sequencing
